# Supplementary material for: HIVprotI: an integrated web based platform for prediction and design of HIV proteins inhibitors
Source: J Cheminform. 2018 Mar 9;10:12. doi: 10.1186/s13321-018-0266-y (PMC5845081; doi:10.1186/s13321-018-0266-y)
Supplement: Supplementary file 2 — Additional file 2. Source code of HIVProtI web server. [file 13321_2018_266_MOESM2_ESM.zip › HIVprotI_Source-code/hivprotI/weka-3-6-6/doc/weka/filters/supervised/attribute/NominalToBinary.html]

NominalToBinary


---


|  |  |  |
| --- | --- | --- |
| **Overview** \| **Package** \| **Class** \| **Tree** \| **Deprecated** \| **Index** \| **Help** \| **Weka's home** | |  |
| **PREV CLASS**   **NEXT CLASS** | **FRAMES**    **NO FRAMES**     **All Classes** |  |
| SUMMARY: NESTED \| FIELD \| CONSTR \| METHOD | DETAIL: FIELD \| CONSTR \| METHOD |  |


---


## weka.filters.supervised.attribute Class NominalToBinary

```
java.lang.Object
  weka.filters.Filter
      weka.filters.supervised.attribute.NominalToBinary
```

**All Implemented Interfaces:**: java.io.Serializable, CapabilitiesHandler, OptionHandler, RevisionHandler, TechnicalInformationHandler, SupervisedFilter

---

``` public class NominalToBinary extends Filter implements SupervisedFilter, OptionHandler, TechnicalInformationHandler ```

Converts all nominal attributes into binary numeric attributes. An attribute with k values is transformed into k binary attributes if the class is nominal (using the one-attribute-per-value approach). Binary attributes are left binary, if option '-A' is not given.If the class is numeric, k - 1 new binary attributes are generated in the manner described in "Classification and Regression Trees" by Breiman et al. (i.e. taking the average class value associated with each attribute value into account)
